# Supplementary material for: Functional Conservation and Divergence of MOS1 That Controls Flowering Time and Seed Size in Rice and Arabidopsis
Source: Int J Mol Sci. 2022 Nov 3;23(21):13448. doi: 10.3390/ijms232113448 (PMC9655188; doi:10.3390/ijms232113448)
Supplement: Supplementary file 1 [file ijms-23-13448-s001.zip › ijms-1900509-supplementary.pdf]

Supplemental Table S1 Primers used in this study

Name	Sequence 5' to 3'	Purpose
OsMOS1target1-BsF	AATAATGGTCTCAGGCGTTGATCCTC CACCAGAAGA	CRISPR/Cas9 vector construction
OsMOS1target1-F0	GTTGATCCTCCACCAGAAGAGTTTTA GAGCTAGAAATAGC	
OsMOS1target2-R0	TCCCGGGTACAAGTCTGCCCGCTTCT TGGTGCC	
OsMOS1target1-BsR	ATTATTGGTCTCTAAACTCCCGGGTA CAAGTCTGCC	
OsMOS1target1-F	GTTATACTTATCACTTGCTTATG	CRISPR/Cas9 mutant genotyping
OsMOS1target1-R	AATAGTTCTGTTCTAATGGC	
OsMOS1target2-F	AACAAGGAAAGAGCATAAC	
OsMOS1target2-R	ACATGTACTCTATCAGATTCG	
OsMOS1-BamHI/F	TTACTTCTGCACTAGGTACCATGACC GTTCTTGGAAAAATTCC	OE:OsMOS1 vector contruction
OsMOS1-KpnI/R	GAATTCCCGGGGATCCTTATTCATCT CGGTAAGAGTTAG	
OSMOS1-RT-F	ATGACCGTTCTTGGAAAAATTCCAA AAC	qPCR
OsMOS1-RT-R	GGTTTGCTACCCCATGTAAGGGTGC CCT	
OsActin1-F	AGGAAGGCTGGAAGAGGACC	
OsActin1-R	CGGGAAATTGTGAGGGACAT	
OsHd1-F	GGCGTCAGTGCTTACACACATT	
OsHd1-R	TCCAGCAGGTGTCAGGATTCT	
OsEhd1-F	ATGGCTCGTGCTACTCCG	
OsEhd1-R	GTTTGACCTTCATTATCCCTA	
OsHd3a-F	GCTCACTATCATCATCCAGCATG	
OsHd3a-R	CCTTGCTCAGCTATTTAATTGCATAA	
OsRFT1-F	TGACCTAGATTCAAAGTCTAATCCTT	
OsRFT1-R	TGCCGGCCATGTCAAATTAATAAC	
AtFLC-qRT-F:	ACTAGAGCCAAGAAGACCGAAC	
AtFLC-qRTR:	ACTAGAGCCAAGAAGACCGAAC	
AtUFP -F:	CTTCTGCTCCCACATGATGATT	
AtUFP -R:	CCAGCAGACATGGAGGTTTTGGGG	
Mos1-F	TGTTGTCTGTCATTTCTTGGCCAGT	For genotyping the OsMOS1 transgenic plants in Arabidopsis
Mos1-R	AGCACGACAGGAGATCGAAGC	

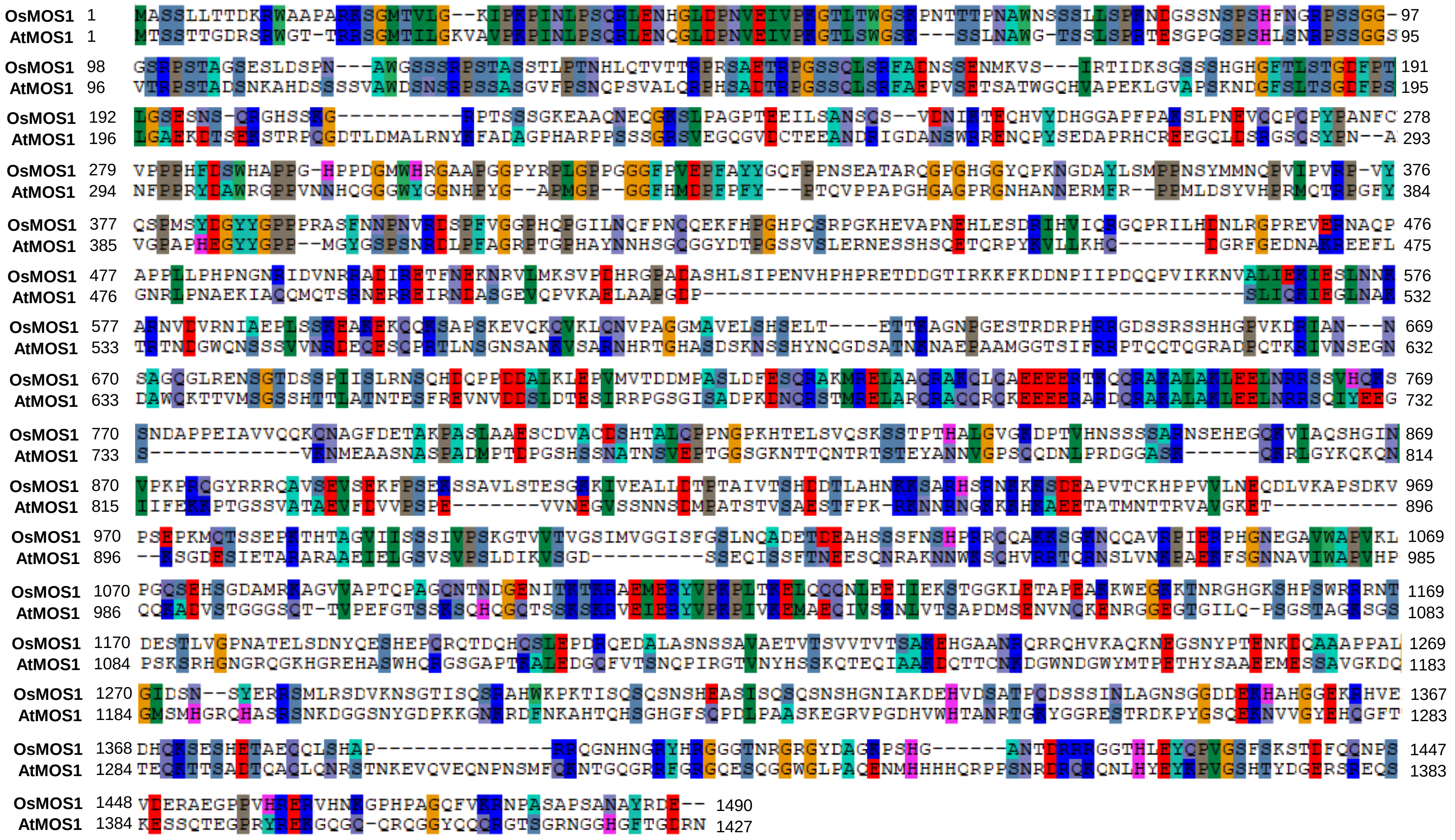

Supplemental Figure S1. The protein sequence alignment of AtMOS1 and OsMOS1. The sequence alignment of full length MOS1 proteins from rice and Arabidopsis were carried out using the MEGA5.02 program (http://www.megasoftware.net/history.php) with default parameters and then displayed on GeneDoc (http://www.softpedia.com/get/Science-CAD/GeneDoc.shtml).

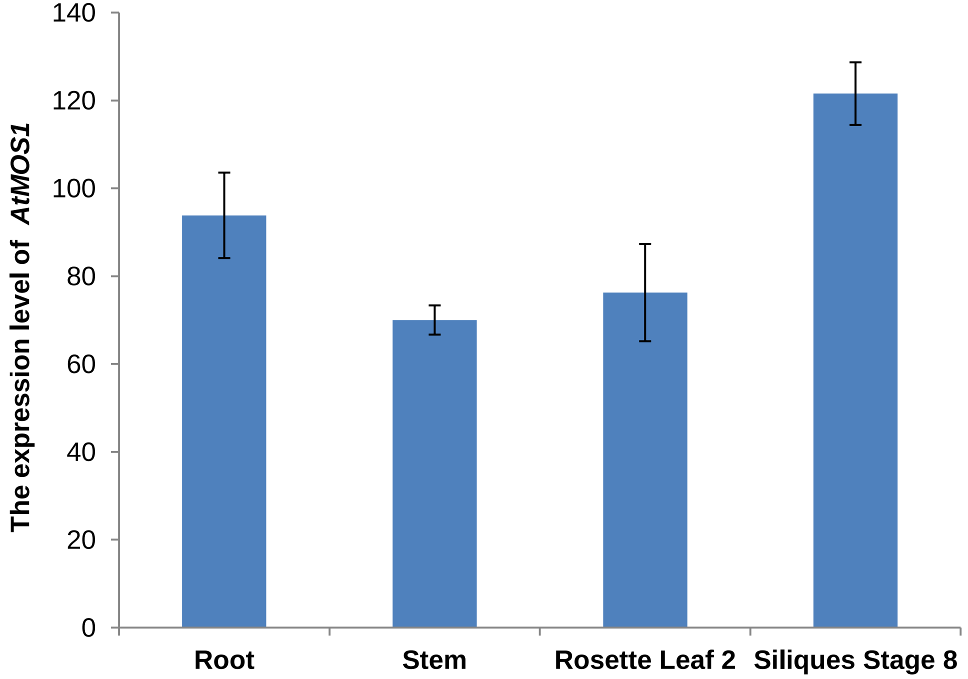

Supplemental Figure S2. The expression profiles of *AtMOS1*. (a) The expression level of *OsMOS1* in different *Arabidopsis* tissues. The data is obtained from the BAR website (http://www.bar.utoronto.ca/). The development datasets correspond to microarray data derived from organs at various stages of development under LD conditions. Error bars indicate standard deviation of three replicates.

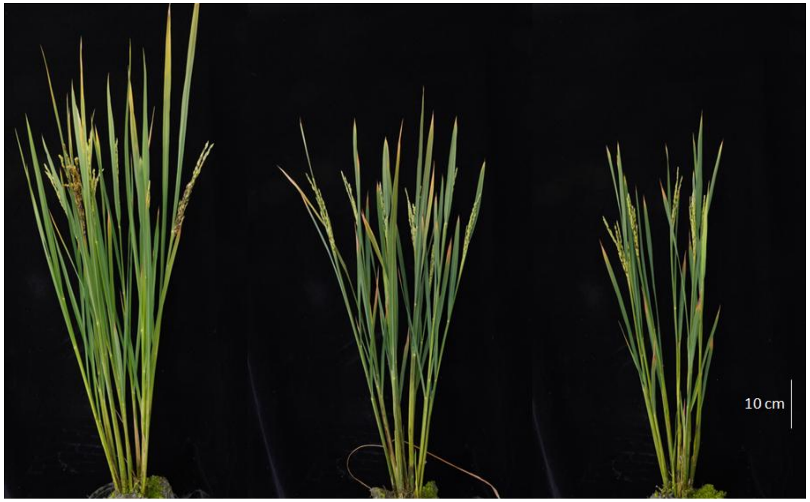

R7954 ***osmos1-4***

Supplemental Figure S3. Late heading phenotypes of *osmos1-4* mutants under NLD conditions. Late heading phenotypes of *osmos1-4* mutants under NLD conditions. Bars = 10 cm.

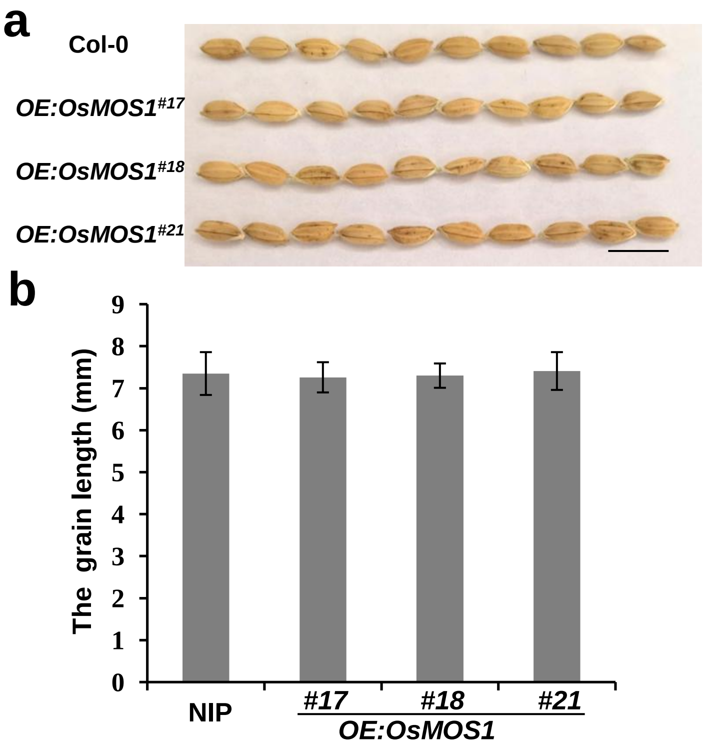

Supplemental Figure S4. Analyses of grain size of NIP and *OsMOS1* overexpression lines
(a) Grain morphology of NIP and *OsMOS1* overexpression lines. Scale bars correspond to 1 cm.
(b) Statistical analysis of grain length and grain width between NIP and *OsMOS1* overexpression lines. Error bars indicate standard deviation, n = 50.

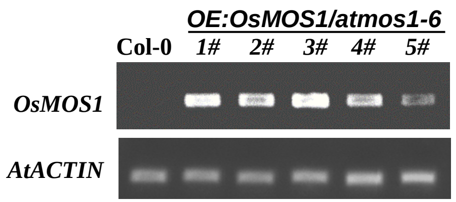

Supplemental Figure S5. RT-PCR analyses of expression of the OsMOS1 genes in wild-type Arabidopsis (ecotype Col-0) and OE:OsMOS1/*atmos1-6* (lines 1, 2, 3, 4, and 5) plants.
